# Quantifying Preferences for CAR‐T Compared to Standard of Care as a First‐Line Treatment Among Patients With Multiple Myeloma

**DOI:** 10.1002/cam4.71072

**Published:** 2025-07-19

**Authors:** Jessie Sutphin, Thomas W. LeBlanc, Ellen Janssen, Laura Hester, Matthew J. Wallace, F. Reed Johnson, Shelby D. Reed

**Affiliations:** ^1^ Duke Clinical Research Institute Duke University School of Medicine Durham North Carolina USA; ^2^ Duke Cancer Institute Duke University School of Medicine Durham North Carolina USA; ^3^ Janssen Research and Development Raritan New Jersey USA

**Keywords:** benefit–risk, CAR‐T, discrete‐choice experiment, multiple myeloma, patient preferences

## Abstract

**Background:**

CAR‐T therapy is approved for the treatment of relapsed refractory multiple myeloma (MM) and is being studied for newly diagnosed MM (NDMM). The use of novel therapies in early‐line MM raises questions on the acceptability of upfront risks in exchange for extended relapse‐free periods without the treatment burden and limitations on daily activities associated with maintenance therapy.

**Methods:**

A discrete‐choice experiment was designed to elicit adults' preferences for hypothetical NDMM treatments. Benefits included time to relapse and reduction of treatment impact on daily activities. Severe adverse events were included to better understand patient preferences for rare but significant events.

**Results:**

On average, extending the time to relapse from 3 years (with moderate limitations on daily activities) to 5 years (without limitations) was three times more important than avoiding a 20% risk of hospitalization due to severe ICANS/CRS. Analysis revealed three latent preference classes: a benefit–risk trading class (65%), a class (28%) unwilling to accept increases in short‐term treatment‐related mortality, and a class (7%) that provided statistically uninformative data. For the trading class, for two additional relapse‐free years with minor limitations, all else equal, patients would accept up to a 30% risk of severe ICANS/CRS‐related hospitalization along with 0% risk of treatment‐related mortality. Alternatively, they would accept up to an 8% risk of treatment‐related mortality with a 0% risk of severe ICANS/CRS‐related hospitalization, or various combinations of lower AE risks.

**Conclusion:**

These results reveal preference heterogeneity among MM patients and the importance of effective communication about the benefits and risks of novel therapies.

## 1 Introduction

Patients newly diagnosed with multiple myeloma (MM) who are ineligible for stem‐cell transplantation (SCT) often receive multi‐agent induction therapy with a combination of proteasome inhibitors, immunomodulatory agents, steroids, and/or anti‐CD38 antibodies, followed by maintenance therapy. However, treatment options for people newly diagnosed with MM are expanding. B‐cell maturation antigen‐directed (anti‐BCMA) chimeric antigen receptor T‐cell therapies (CAR‐T), initially approved for patients who have relapsed after four or more lines of treatment, are now available as second‐line or third‐line therapy for select patients [1, 2, 3, 4, 5]. Findings indicate the potential for a durable, long‐term response without maintenance therapy for some patients receiving CAR‐T as a second or later line treatment [6, 7, 8]. Additional studies are investigating anti‐BCMA CAR‐T (hereafter, CAR‐T) in earlier lines of treatment, including use as a first‐line therapy in patients newly diagnosed with MM who are ineligible for SCT.

There are benefits and potential risks associated with any MM treatment option. With expanding treatment options, some patients will face a choice between upfront treatment‐related risks or ongoing maintenance therapy with associated long‐term impacts on daily health‐related quality of life. Patients receiving CAR‐T therapy may experience up‐front treatment‐related risks in the first few months following apheresis [4]. However, after the initial treatment period, these patients can experience extended relapse‐free periods during which they will avoid ongoing treatment burden and risks that are associated with maintenance therapy. Whether patients choose CAR‐T over maintenance therapy early in the course of their disease will depend on whether they perceive that the possibility of a longer relapse‐free and treatment‐free interval outweighs the possibility of potential upfront CAR‐T related adverse events. Understanding patients' acceptance of these benefit–risk tradeoffs will provide valuable information to stakeholders seeking to incorporate patient perspectives in decision making. The objective of this study was to quantify patients' preferences about benefits and risks of first‐line treatment options for people newly diagnosed with multiple myeloma (NDMM), including CAR‐T and conventional therapy.

## 2 Methods

### 2.1 Survey Development

The survey consisted of three sections: one in which respondents were trained on how to complete the discrete choice experiment (DCE) (including comprehension questions), the DCE itself, and questions on their medical history and demographics.

### 2.2 Discrete Choice Experiment

A DCE was designed to elicit treatment preferences in the setting of newly diagnosed MM. DCEs are widely used to elicit patients' treatment preferences and to quantify risk tolerance [9]. In a DCE, patients answer a series of questions presenting choices between two or more hypothetical treatment profiles defined by treatment features or attributes. The attributes are represented by different levels which are systematically varied across hypothetical treatment profiles. The attributes in this DCE represent primary benefits and potential adverse events associated with CAR‐T or maintenance therapy. Selection of the attributes was informed by expert opinion, discussions with patient stakeholders, and clinical literature available at the time, primarily early‐phase clinical trial results with CAR‐T in heavily pre‐treated patient populations [3, 10]. The attributes included clinical outcomes that patients with newly diagnosed MM would consider when making treatment choices, including rare but severe potential AEs. This approach is common in preference studies, as a method to identify a patient's maximum risk tolerance, thus the survey includes some hypothetical treatment profiles with risk levels higher than supported by evidence.

Treatment benefit for the hypothetical treatment profiles was presented as relapse‐free time (3, 5, and 10 years). Ongoing treatment burden was presented as limits on daily activities due to treatment side effects (no limits, minor limits, and moderate limits). Minor and moderate limits on daily activities represent impacts from side effects patients might experience with ongoing maintenance therapy such as fatigue, neuropathy, and gastrointestinal problems [11, 12]. The survey text defined these limits and provided examples (see Supporting Information S1). With minor limits, patients could continue to do all their usual activities but with less energy, while with moderate limits, they would not be able to do all their usual daily activities. The no‐limits level corresponds to treatment‐free time following CAR‐T. Levels for time until relapse apply to both maintenance therapy and CAR‐T [13, 14].

Although upwards of 90% of patients with heavily pre‐treated MM receiving CAR‐T cell therapy experience CRS, prophylactic management and supportive care have been shown to reduce its incidence and severity [15, 16]. In this study, the attribute for chance of severe ICANS/CRS‐related hospitalization in the first 3 months of treatment (hereafter hospitalization risk) represents the incidence of these adverse events with grade 3 or 4 severity and was described as brain or immune‐system related complications severe enough to require a 10‐day hospitalization with residual signs and symptoms lasting 2 months. Levels for this attribute ranged from 0% to 20% to purposefully span the clinically relevant and plausible ranges.

The final attribute for the hypothetical treatment profiles was the chance of complications that lead to death within the first 3 months of treatment. Treatment‐related mortality is rare, but is the most severe potential AE of any MM therapy. In this preference study, lower levels for treatment‐related mortality risk (i.e., 0% and 1%) are consistent with incidence rates among those receiving maintenance therapy or CAR‐T. The higher levels for treatment‐related mortality risk selected for this study (i.e., 5% and 10%) were intentionally chosen to be higher than treatment‐related mortality rates reported for any therapy in early line to ensure the survey would identify a maximum risk tolerance without extrapolating beyond the highest risk levels shown [11].

Combinations of DCE attribute levels that define each hypothetical treatment profile in the DCE choice questions (see Supporting Information S1) are defined by an experimental design with known statistical properties and are not based on treatment profiles that are available or currently under development. The design requires that patients evaluate tradeoffs among desirable and undesirable treatment features. The design generated for this study included 144 choice questions split into 18 blocks of eight choice questions each. Each patient answered one block of eight questions so that all blocks were approximately equally represented. Additionally, the order of the choice questions in each block was randomized to avoid potential order effects.

### 2.3 Choice Context and Training Materials

The survey provided an explanation of the choice context, which is the hypothetical clinical scenario to motivate the need to choose among treatment options. Most patients invited to complete the survey had already received initial treatment for MM, many before CAR‐T was an option. Therefore, to create a common baseline scenario and to reduce cognitive bias, the hypothetical choice context for the study used third‐person voice [17, 18]. Patients were asked to suppose that they had a friend who recently was diagnosed with MM, who was their same gender, about the same age, and similar in terms of daily activities and outlook on life. Patients were asked to choose the treatment they thought would be best for their friend as a proxy for what they would choose for themselves if they were in the same situation. Additionally, they were told their friend is not eligible for SCT, has begun their first phase of induction therapy, and now they need to choose a treatment that would begin after they complete 4–6 months of induction therapy. The DCE treatment profiles were labeled as “Treatment A” and “Treatment B” to avoid effects stemming from possible positive or negative attitudes patients may have towards “CAR‐T” or “maintenance therapy” labels.

Following best practices for stated‐preference survey design, the survey instrument included low reading‐level descriptions of each attribute (see Supporting Information S1), content providing training on attributes, presentation of risks using icon arrays, choice‐task layout, and the hypothetical decision context [19]. Patients also were offered a choice whether they would like to receive training on the choice‐task layout via an informational video or a series of still images. Eight comprehension questions were designed to test on and reinforce the information provided.

### 2.4 Pretesting

The survey instrument was pretested between 21 April 2022 and 8 July 2022 in a series of one‐on‐one virtual interviews with 10 adults with MM. During the interviews, patients were asked to read the survey content aloud and to describe their thinking when responding to survey items. The research team also systematically varied the attribute levels shown in the treatment profiles to gauge when participants would switch to the alternative treatment option. These discussions informed the final attribute levels selected for the DCE. The final survey instrument was programmed for online administration using Lighthouse Studio version 9.14.0 (Sawtooth Software Inc., Provo, UT).

### 2.5 Study Sample and Recruitment

The sampling frame included 1139 adults with a diagnosis of MM identified using the Duke Cancer Institute (DCI) Tumor Registry, which includes patients who were treated at the DCI at any point in their disease and are known to be alive at the last point of contact. As a result, the sample included patients who were in remission and patients with relapsed‐refractory disease who had a variety of treatment experiences. Most patients in the registry lived in the mid‐Atlantic region of the US. Eligible patients were sent mailed letters inviting them to participate in the study. The letter included a web address and a patient‐specific alphanumeric passcode that provided access to the survey.

The study protocol was approved by the Duke University Health System Institutional Review Board for Clinical Investigations (Pro00111815). All patients provided informed consent prior to their participation in pretest interviews or completing the survey. Patients who participated in pretest interviews were offered $100, and patients completing the online survey were offered $60.

### 2.6 Statistical Analysis

#### 2.6.1 Descriptive Statistics

Descriptive statistics summarize sociodemographic characteristics for the full cohort of eligible patients and for the subset who completed the survey. Descriptive statistics also include additional information collected for the study sample.

Some patients always choose the treatment alternative with the lower treatment‐related mortality or hospitalization risk. This type of choice pattern was evaluated by summarizing the number of times patients chose the treatment options with lower risks across choice questions. For strongly risk‐averse patients, larger benefit levels offered were never sufficient to compensate for higher risk levels. However, we cannot rule out the hypothesis that some patients chose the lower risk as a simplifying decision heuristic to avoid the effort of evaluating benefit–risk tradeoffs.

#### 2.6.2 Analysis of Choice Data

Appropriate analysis of choice data accounts for the pattern(s) of choices observed. Therefore, rather than pre‐specifying a model prior to data collection, an exploratory analysis was undertaken to specify the final model. Exploratory analysis began with conditional‐logit and random‐parameters logit models, as described in Supporting Information S1.

Latent‐class analysis (LCA) identifies unique preference‐weight estimates for each of a pre‐specified number of classes, where each class represents a distinct preference pattern observed in the responses. LCA began with an investigation into the quality of choice responses. In a two‐class model, all preference parameters were restricted to be zero for one of the two classes to identify patients that provided no useful information to model preferences (i.e., randomly selected treatment options). Covariates characterizing potential validity issues included the number of comprehension questions answered incorrectly, failure of a within‐set dominated‐choice question (occurs when a respondent chooses a treatment option that is worse in all respects than an alternative option), strong disagreement with the reliability of their responses to the DCE questions, requiring help with written health materials (i.e., poor health literacy), and the patient's subjective numeracy score [20, 21] (see Table 1 for summary statistics. Debriefing questions are shown in Supporting Information S1). Only 7% of patients had responses consistent with the uninformative class (i.e., random responses). Those with more incorrect responses to comprehension questions and those who disagreed that they would make the same choices if they took the survey again were more likely to be members of the uninformative class. The other covariates were not associated with membership in this class.

**TABLE 1:** Patient characteristics.

	Study sample (*n* = 176)	Cancer registry (*n* = 1137)
Age (years)		
Mean (SD)	65.9 (9.4)	60.3 (10.4)
Female	89 (50.6%)	536 (47.1%)
Hispanic ethnicity	4 (2.3%)	24 (2.1%)
Race^a^		
American Indian or Alaskan Native	5 (2.8%)	8 (0.7%)
Asian	4 (2.3%)	11 (1.0%)
Black or African American	38 (21.6%)	363 (31.9%)
White	131 (74.4%)	698 (61.4%)
Other	2 (1.1%)	15 (1.3%)
Prefer not to answer	1 (0.6%)	42 (3.7%)
Highest level of education completed		
High school or less	36 (20.5%)	
Technical school or 2‐year college degree	22 (12.5%)	
4‐year college degree or more	118 (67.0%)	
Employment		
Retired	100 (56.8%)	
Employed full‐ or part‐time	43 (24.4%)	
Disabled/not able to work	29 (16.5%)	
Homemaker	8 (4.6%)	
Unemployed	1 (0.6%)	
Other	2 (1.1%)	
Time since MM diagnosis (years)		
Mean (SD)	5.6 (3.6)	
Have ever had a MM relapse	54 (30.7%)	
Ever had bone marrow or stem‐cell transplant for MM	141 (80.1%)	
No. of incorrect responses to comprehension questions (out of eight)		
Mean (SD)	0.57 (0.78)	
Incorrect response to within‐set dominated‐choice question	10 (5.7%)	
Requires help with written health materials	33 (18.8%)	
3‐item subjective numeracy score		
Mean (SD)	4.9 (1.2)	

*Note: p*‐values are not provided because the samples are not independent.

^a^ Patients could choose more than one option; thus, percentages do not add up to 100%.

Next, LCA models were estimated for two to four classes without modeling constraints. Information criteria statistics and inspection of qualitative differences across classes initially indicated the data were best fit by a 2‐class model. This method of LCA is known as an inductive approach, where all attribute parameters are unconstrained and without priors (i.e., entirely data driven). In the unconstrained 2‐class model, one class (53% membership probability) had significant preference weights only for the treatment‐related mortality‐risk attribute levels. Preference weights for the other attribute levels were not significantly different from each other. The second class (47%) had significant preferences for all study attributes, indicating that patients were trading off benefits and risks (i.e., trading preferences). Both classes had significant preferences for lower treatment‐related mortality risk, but the classes varied in the relative importance they placed on other study attributes.

For the final LCA model, we adopted a deductive approach. With this approach, we used insights gained from the inductive modeling approach to represent choice behaviors observed in the data. The final model included three classes. One unconstrained class was specified to model preferences consistent with acceptance of tradeoffs across all benefits and risk attributes (benefit–risk trading class). A second class was specified to represent preferences consistent with minimizing treatment‐related mortality risk regardless of benefits offered (treatment‐related mortality‐risk minimizing class). In the model specification for this class, all attribute parameters other than treatment‐related mortality‐risk were constrained to zero. Such preferences provide no statistical information on the relative importance of other attributes. A third class was included to model responses that provided no useful statistical information about preferences (i.e., uninformative class). To test whether patient characteristics were associated with class membership, the following prespecified covariates were included: comorbidities, race, education, age, whether a patient had a relapse or a transplant, and time since their MM diagnosis. Age and time since MM diagnosis were modeled as continuous covariates.

Additionally, the final LCA model provides three class‐specific individual membership probabilities for each patient. For each class, a probability close to 100% indicates a high probability that a patient's preferences are accurately represented by the estimated preference weights for that class. We report the proportion of the sample assigned to each latent class based on the highest individual class‐membership probability and the proportion of classification errors. A low classification error statistic indicates that classes are well separated and a high degree of confidence in individual modal class assignments.

Categorical attribute levels were effect‐coded with interactions between time to relapse and limits on daily activities. Effect‐coding generates preference‐weight estimates and *p*‐values for all attribute levels, each of which is interpreted relative to the mean preference weight for that attribute, rather than in comparison to an omitted attribute level (as with dummy‐coding). Preference‐weight estimates are thus centered around zero for each attribute.

Descriptive statistics of patient characteristics were summarized using Stata 17 (StataCorp LLC, College Station, TX). Choice data from the DCE were analyzed with Stata 17 and Latent GOLD software (Statistical Innovations Inc., Belmont, MA). Estimates of risk‐tolerance were calculated for the benefit–risk trading class.

## 3 Results

### 3.1 Demographics and Comprehension Questions

A total of 194 patients, 17% of the 1139 who were mailed invitation letters, accessed the online survey between 19 November 2022 and 4 February 2023. Six patients were ineligible based on their answers to the screening questions, and 12 did not complete the survey. The final analysis sample included 176 completed surveys with a median completion time of 32 min. The demographic characteristics available from the tumor registry for patients with MM who were mailed a study invitation revealed that the final survey sample was slightly older but similar in terms of gender and ethnicity (Table 1).

Slightly more than half (57%) of patients answered all eight comprehension questions correctly. The mean number of correct responses on the comprehension questions was 7.4 (SD 0.9) out of 8. Approximately two‐thirds (76%) of patients elected to see a series of still images rather than a video in the training section of the survey instrument.

### 3.2 DCE Choice Patterns

Fifty‐one percent of the sample chose the hypothetical treatment profile with a lower chance of death in at least six of the eight DCE questions, including 10 patients who chose the treatment option with the lower treatment‐related mortality risk in all eight DCE questions regardless of the level of benefit offered. A relatively small proportion of the sample (13%) chose the treatment with the lower chance of hospitalization in at least six of the eight DCE questions.

### 3.3 Latent‐Class Analysis

Most patients (86%) had an individual membership probability of 80% or higher for one of the three classes in the final model, meaning that they had an 80% or higher probability of belonging to their assigned class. Across the three classes, the average classification error statistic for the model was 8%. The results indicate a high degree of certainty that patients were correctly assigned to a latent class that best represents their treatment preferences.

**FIGURE 1:**
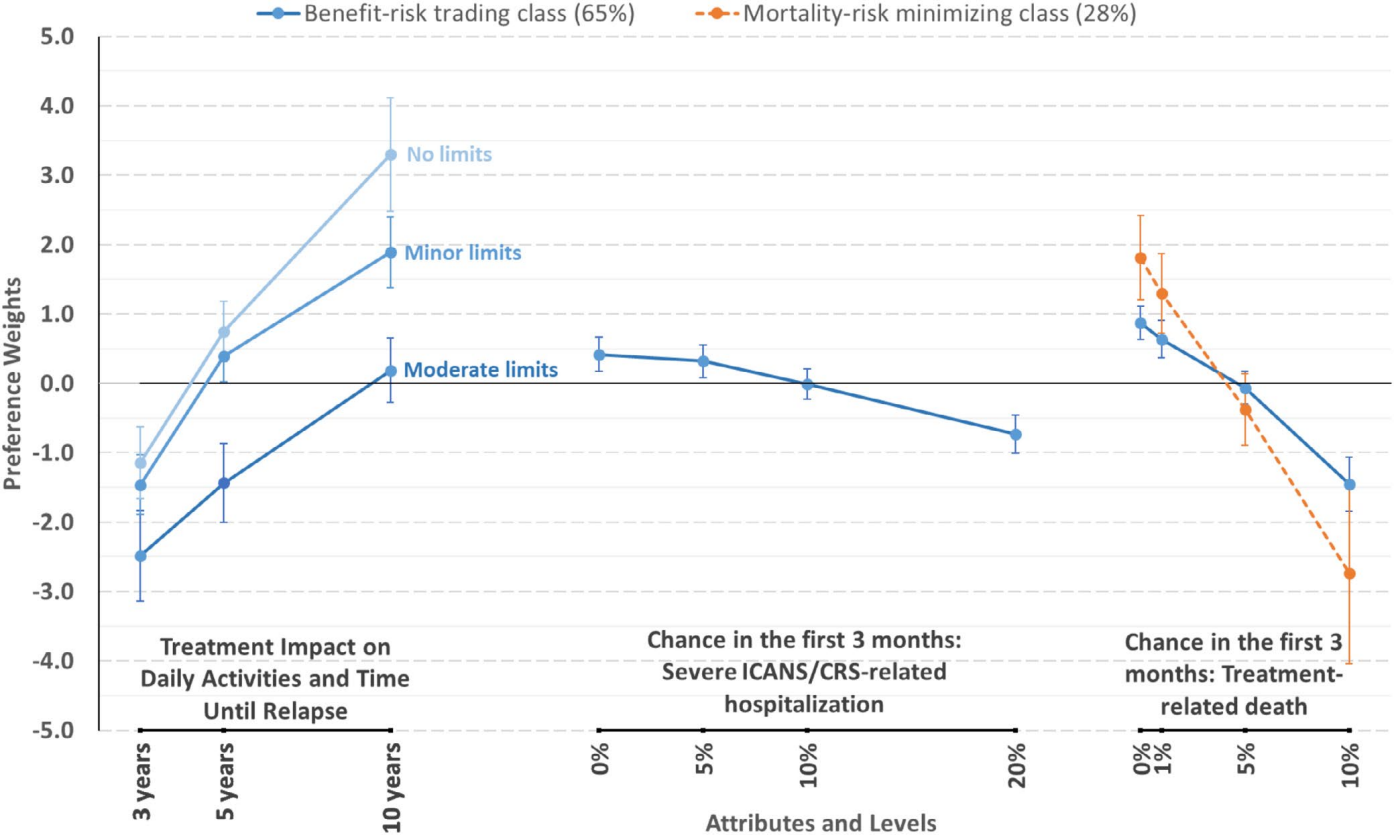
Relative preference weights (*N* = 176). Error bars are the 95% confidence intervals around the point estimates. Percentages in parentheses are the sample‐level membership probability for each class. The uninformative class (7%) is not plotted as all attribute parameters for that class were constrained to be zero. The attribute for severe ICANS (immune effector cell‐associated neurotoxicity syndrome) and severe CRS (cytokine release syndrome) represents the incidence of these adverse events with grade 3 or 4 severity and is described as brain or immune‐system‐related complications severe enough to require a 10‐day hospitalization with residual signs and symptoms lasting 2 months.

The benefit–risk trading class represents preferences for 65% of the sample. As shown in Figure 1 and Table 2, patients predicted to be in this class were willing to accept risks for longer time to relapse and/or fewer impacts on daily activities. Preferences for this class were logically ordered for all attributes, indicating that lower levels of risk were preferred to higher levels, more time until relapse was preferred to less time, and no or minor limits on daily activities were preferred to moderate limits.

**TABLE 2:** Results from latent‐class analysis (*N* = 176).

Attributes	Levels		Benefit–risk trading class (65%)			Treatment related mortality‐risk minimizing class (28%)			Uninformative class (7%)		
			*β*	SE	*p*	*β*	SE	*p*	*β*	SE	*p*
Treatment impact on daily activities and time until relapse	None	10 years	3.297	0.417	0.000	0.000	—	—	0.000	—	—
		5 years	0.743	0.222	0.001	0.000	—	—	0.000	—	—
		3 years	−1.140	0.264	0.000	0.000	—	—	0.000	—	—
	Minor	10 years	1.890	0.260	0.000	0.000	—	—	0.000	—	—
		5 years	0.397	0.192	0.040	0.000	—	—	0.000	—	—
		3 years	−1.459	0.218	0.000	0.000	—	—	0.000	—	—
	Moderate	10 years	0.191	0.237	0.421	0.000	—	—	0.000	—	—
		5 years	−1.439	0.290	0.000	0.000	—	—	0.000	—	—
		3 years	−2.480	0.333	0.000	0.000	—	—	0.000	—	—
Chance in the first 3 months: 10‐day Severe ICANS/CRS‐related hospitalization	0%		0.419	0.124	0.001	0.000	—	—	0.000	—	—
	5%		0.321	0.120	0.008	0.000	—	—	0.000	—	—
	10%		−0.006	0.111	0.957	0.000	—	—	0.000	—	—
	20%		−0.734	0.139	0.000	0.000	—	—	0.000	—	—
Chance in the first 3 months: treatment‐related death	0%		0.876	0.123	0.000	1.814	0.310	0.000	0.000	—	—
	1%		0.636	0.139	0.000	1.296	0.292	0.000	0.000	—	—
	5%		−0.062	0.120	0.608	−0.375	0.265	0.159	0.000	—	—
	10%		−1.450	0.199	0.000	−2.735	0.664	0.000	0.000	—	—
Covariates											
4 or more comorbidities			−0.115	0.421	0.786	0.155	0.673	0.819	−0.040	0.516	0.939
Non‐White race			−0.400	0.352	0.258	0.636	0.505	0.210	−0.236	0.380	0.536
College degree or higher			**0.862**	**0.424**	**0.044**	**−1.396**	**0.643**	**0.031**	0.534	0.396	0.178
Age (years)			**−0.052**	**0.018**	**0.005**	**0.076**	**0.026**	**0.004**	−0.023	0.018	0.185
History of MM relapse			0.669	0.424	0.117	−0.232	0.711	0.744	−0.436	0.499	0.383
History of stem‐cell transplant			0.276	0.486	0.572	0.712	0.623	0.255	**−0.988**	**0.485**	**0.043**
Years since MM diagnosis			−0.026	0.074	0.725	−0.109	0.099	0.271	**0.135**	**0.066**	**0.043**

*Note:* Numbers reported in parentheses are membership probabilities for each class. Numbers reported with bold text indicate statistical significance (i.e., *p* < 0.05). Attribute levels in this model are effect‐coded. Estimates are interpreted relative to the mean preference weight for each attribute, rather than in comparison to an omitted attribute level (as with dummy‐coding). Preference‐weight estimates are thus centered around zero for each attribute. Age and years since MM diagnosis were modeled as continuous covariates.

Differences in preference weights between two levels for an attribute indicate the relative importance of those differences. Preference weights for 0%, 5%, and 10% hospitalization risk were not different from each other, but all three were statistically significantly different from 20%, indicating that respondents were relatively unconcerned about the risk of hospitalization due to severe ICANS/CRS until the risk level was higher than 10%. A 7‐year change in relapse‐free time with no limitations on daily activities was most important to respondents compared to differences between levels within other attributes. A 20% change in chance of hospitalization was least important. A change from 3 years until relapse with moderate limits to 5 years without any limits was nearly three times more important than avoiding a 20% risk of hospitalization for severe ICANS/CRS. Avoiding a 10% chance of treatment‐associated death was about equally important as gaining 7 additional years until relapse with no limits on daily activities.

Twenty‐eight percent of the sample chose hypothetical treatment alternatives based solely on treatment‐related mortality risk. These patients were unwilling to accept hypothetical increases in treatment‐related mortality risk for additional time until relapse or to avoid limits on daily activities. In addition, 7% of the sample made choices that were statistically uninformative about their preferences.

Younger patients with a 4‐year college degree were significantly more likely to accept benefit–risk tradeoffs, while older patients without a 4‐year college degree were significantly more likely to demonstrate treatment‐related mortality risk minimizing preferences. Patients who had not received a transplant and who had a longer history of MM were more likely to provide statistically uninformative choice data (Table 2).

#### 3.3.1 Risk‐Tolerance

The ratio of a change in benefit to a one‐unit change in risk indicates how much increase in risk is equivalent to a specified increase in benefit, or the maximum‐acceptable risk (MAR) for a defined benefit, all else equal. Preference weights were used to calculate the simultaneous MAR thresholds for short‐term treatment‐related mortality and hospitalization risks that patients would accept in exchange for two additional years until relapse with differing levels of daily‐activity limitations [22, 23].

**FIGURE 2:**
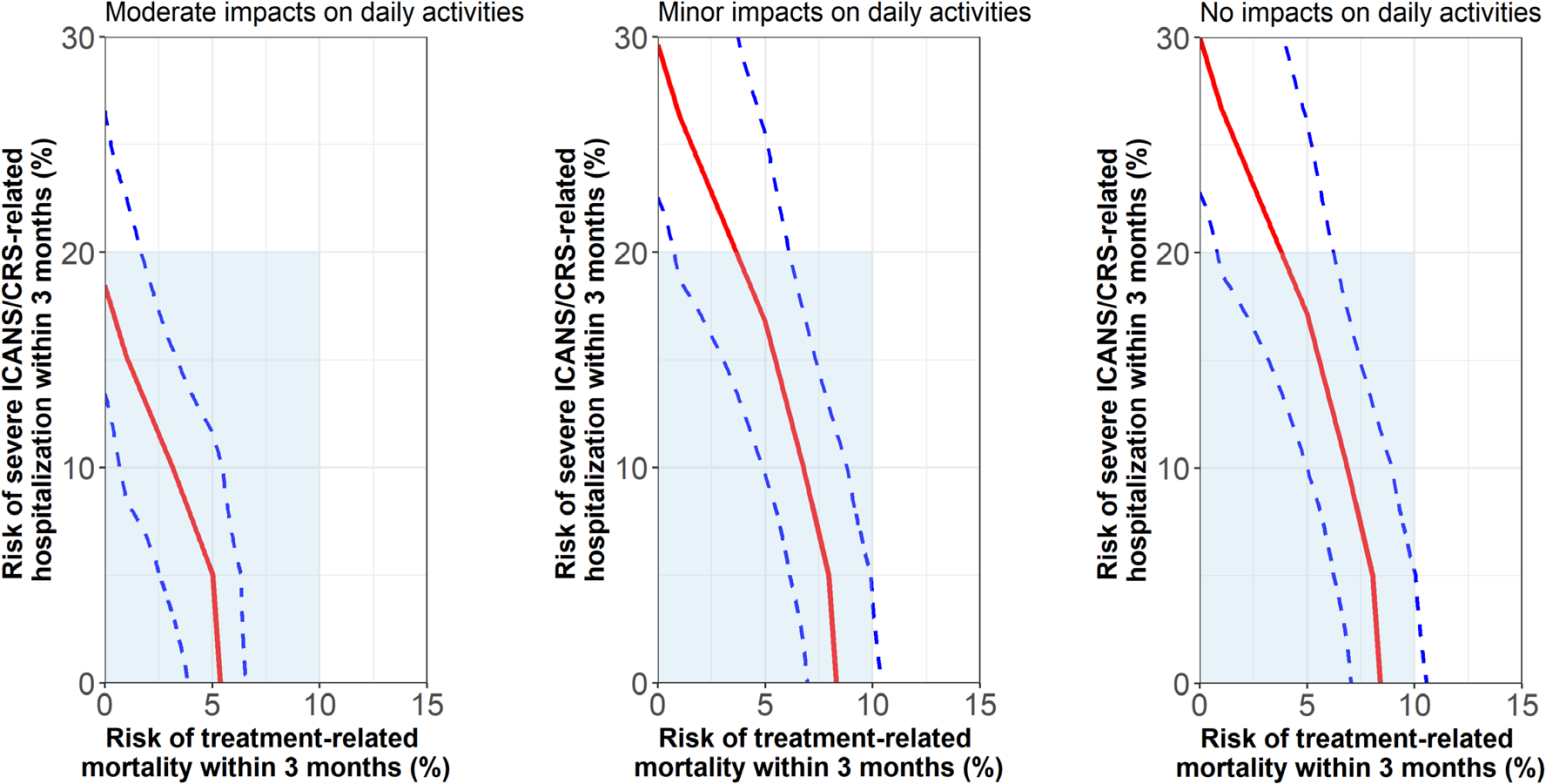
Simultaneous maximum‐acceptable risk for two additional years until relapse across levels of limitations on daily activities—benefit risk trading class (65%). The benefit–risk trading class choice patterns indicate acceptance of some increases in treatment‐related mortality risk and severe ICANS/CRS related hospitalization risk for 2 additional years until relapse. The shaded area represents the ranges covered by risk levels included in the study. The blue dashed lines are the 95% confidence intervals.

As MARs require tradeoffs to estimate, simultaneous MARs for increases in time to relapse from three to 5 years were only calculated for the benefit–risk trading class (Figure 2). When limitations on daily activity were minor, in exchange for two additional relapse‐free years, patients accept a maximum risk of severe ICANS/CRS‐related hospitalization of 30% along with a 0% risk of treatment‐related mortality, or they would accept up to an 8% risk of treatment‐related mortality with a 0% risk of severe ICANS/CRS‐related hospitalizations, or various combinations of lower risks of both AEs. When the risk of hospitalization is 5% or less, the maximum acceptable risk of treatment‐related mortality for a gain of two relapse‐free years with moderate limits on daily activities is 5.0% (95% CI: 2.5%–6.4%). With minor activity limits, the maximum acceptable treatment‐related mortality risk increases to 8.0% (95% CI: 6.1%–9.9%). As the risk of hospitalization increases to 20%, the maximum acceptable treatment‐related mortality risk decreases to 0% with moderate activity limits or 3.6% (95% CI: 0.8%–6.1%) with minor limits.

## 4 Discussion

This study utilized a DCE survey to elicit treatment preferences for first‐line therapy for MM among patients recruited from a comprehensive cancer registry of patients in the mid‐Atlantic US with a range of MM and treatment experiences. Previous studies have evaluated patient and clinician preferences for CAR‐T for other cancers [24, 25] and patient, caregiver, and clinician preferences for MM treatments other than CAR‐T [26, 27, 28]. This study is the first to elicit patient preferences for CAR‐T therapy compared to standard of care specifically for MM. Recently available results from the CARTITUDE‐4 [11] and KarMMa‐3 [5] trials highlight the benefit–risk considerations patients and their oncologists will face when considering CAR‐T and other novel therapies in earlier lines of treatment.

Our approach to LCA facilitated the identification of unique and heterogeneous preference patterns in the data controlling for the influence of poor‐quality responses from a small proportion of patients. In this sample, across hypothetical treatment profiles, a majority of patients (65%) accepted higher potential upfront treatment risks for longer relapse‐free time and longer time without treatment‐related limitations on daily activities.

A recent study revealed that severe ICANS/CRS‐related hospitalization risk levels shown in the choice tasks in this study were higher than rates of grade 3 or 4 CRS or ICANS, or treatment‐related mortality observed with CAR‐T treatment or standard of care in earlier lines of therapy [29]. However, patients' responses to these hypothetical choice tasks enabled estimation of the upper limit of patients' risk tolerance, even if that risk is higher than what has been observed in clinical studies and real‐world evidence. Among respondents willing to make benefit–risk tradeoffs, choices across constructed pairs of treatment options indicated that they would accept up to 5% severe ICANS/CRS‐related hospitalization risk (non‐fatal) and 8% treatment‐related mortality risk for two additional relapse‐free years with minor activity limits and up to 5% severe ICANS/CRS‐related hospitalization risk (non‐fatal) and 5% treatment‐related mortality risk for two additional years with moderate limits.

By asking patients to consider the most severe types of AEs (i.e., potential risk of treatment‐related mortality) at risk levels higher than reported in clinical studies, these results provide an understanding of the maximum‐acceptable risk that patients with NDMM might accept at different levels of efficacy with early‐line treatment. If patients in this study were willing to accept the possibility of treatment‐related mortality or hospitalization due to severe AEs, it is reasonable to argue that they would accept less severe treatment‐related risks.

We also examined demographic characteristics associated with class membership. Younger patients with a 4‐year college degree were more likely to belong to the trading class, while older patients without a 4‐year college degree were more likely to belong to the treatment‐related mortality‐risk minimizing class. While we are unable to confirm, we can surmise a few possibilities for these results. Younger patients might perceive themselves to have more to gain from extended relapse‐free time with fewer limits of daily activities than older patients. It may also be that those with less education simplified the choice task by choosing only based on treatment‐related mortality risk, rather than considering both benefits and risks. These findings underscore the importance of following a shared decision‐making process for MM treatment choices in practice.

As with all stated‐preference elicitation methods, there is potential for hypothetical bias. Future patients making actual decisions about treatment alternatives for themselves could make different choices, but there also are important real‐world factors that could influence real‐world treatment decisions. Patients participating in the survey were more likely to be white than in the sampling frame, and they were fairly well educated, with two‐thirds having a college degree. However, by recruiting patients included in the DCI Tumor Registry, our sample includes patients from across the mid‐Atlantic region of the US with a verified physician diagnosis and a variety of disease and treatment experiences. Thus, the sample is likely to be more representative of the MM population than what could have been achieved through other recruitment methods commonly used in survey‐based research.

One limitation of DCEs is that not all possible treatment‐related benefits or potential adverse events can be included in the treatment‐choice questions as it would be too cognitively burdensome for patients. Care was taken to choose the treatment attributes likely to be most important to patients and attributes that were needed to answer the research question. The choice tasks in this study only considered upfront CAR‐T related treatment risks and risks associated with maintenance treatment—they did not consider other potential upfront risks that might be associated with current/SOC treatment of NDMM. Administration features unique to CAR‐T treatment were also excluded.

Results from this preference study can be useful to regulatory decision‐makers weighing the potential benefits to patients of greater durability of treatment benefits but with risks of life‐threatening adverse events. This study demonstrated that, when presented with hypothetical treatment choices, a majority of a diverse sample of previously treated patients were willing to accept increases in short‐term risks for increases in relapse‐free time and decreases in impacts on daily activities.

These results are also useful for clinical practice and highlight the importance of discussing the benefits and risks associated with different MM first‐line treatment options. Understanding the preference heterogeneity that exists among patients with MM motivates the need for shared‐decision making and explicit discussions to understand each patient's tolerance for potential adverse events associated with the different NDMM therapies. As studies investigate the potential for CAR‐T and other novel therapies in earlier lines of treatment with benefit–risk profiles that might differ from traditional therapies, these discussions will become more important.

## Author Contributions

All authors contributed to study conduct, concept and design, and reviewed and revised the manuscript critically for important intellectual content, agreed to submit to the current journal, gave final approval of the version to be published, and agreed to be accountable for all aspects of the work. J.S., M.J.W., F.R.J., and S.D.R. contributed to data acquisition, analysis, and interpretation.

## Disclosure

J.S., T.W.L., M.J.W., F.R.J., and S.D.R. received salary support for work related to this project through a contract research agreement between Duke University and Janssen Research & Development LLC. T.W.L., F.R.J., and S.D.R. report research funding and external relationships at https://scholars.duke.edu/. T.W.L. is a Scholar in Clinical Research of the Leukemia & Lymphoma Society. E.J. and L.H. are employees of Janssen Research & Development and hold stock in Johnson & Johnson.

## Ethics Statement

The study protocol was approved by the Duke University Health System Institutional Review Board for Clinical Investigations (Pro00111815).

## Consent

All patients provided informed consent prior to completing the survey.

## Conflicts of Interest

The authors declare no conflicts of interest.

## Supporting information


Data S1:

## Data Availability

De‐identified data are available from the corresponding author upon receipt of a study plan.
