# Supplementary material for: Quantifying Preferences for CAR‐T Compared to Standard of Care as a First‐Line Treatment Among Patients With Multiple Myeloma
Source: Cancer Med. 2025 Jul 19;14(14):e71072. doi: 10.1002/cam4.71072 (PMC12274656; doi:10.1002/cam4.71072)
Supplement: Supplementary file 1 — Data S1: [file CAM4-14-e71072-s001.docx]

**Supplementary Materials**

**1. Attribute Descriptions**

**How Long the Treatment Worked**

Doctors do not know how much benefit a particular person will get from a treatment for multiple myeloma. However, based on their experience treating large numbers of people, doctors know how long a treatment worked (controlled the disease) for most people. When a treatment stops working for multiple myeloma, it is known as a relapse.

The shorter the time until the first relapse is a sign that a person’s multiple myeloma is more aggressive, and more intense treatments may be necessary. Once a person has had a relapse, future relapses often occur more quickly.

**Side Effects from Treatments and Effects on Daily Activities**

In addition to thinking about how long treatments have worked, possible side effects of treatments could also be important for you to consider for your friend.

Some common side effects of multiple myeloma treatments have been:

- Tiredness or fatigue
- Numbness, tingling, and burning in fingers and toes (neuropathy)
- Nausea or upset stomach

These side effects have varied in severity, and some treatments caused several side effects at the same time. They have affected people’s ability to do their usual daily activities, like working, doing household chores like grocery shopping and preparing meals, and visiting with friends and relatives.

| **No limits** | - Some treatments had no significant side effects and **had** **no impact on people’s daily activities**. - People taking these treatments could still do everything they could do before they were diagnosed with multiple myeloma. |
| --- | --- |
| **Minor limits** | - Some treatments had side effects that **somewhat limited people’s daily activities**. People could continue to do all their usual activities, but with less energy and they tired out more quickly. - People who worked a “desk job” before their diagnosis could continue to work the same hours. But, they were less productive than before. The impact was greater on people who had more physically demanding jobs. - They could shop at the grocery store, cook meals, and do housework. But, these activities took longer than before. |
| **Moderate limits** | - Some treatments had side effects that **more noticeably limited people’s daily activities**. People are **not able** to do **all** their usual daily activities. - People who worked a “desk job” before their diagnosis could continue to work. But, they had to cut back their hours and were less productive than before. People with more physically demanding jobs had to stop working. - They could sometimes shop at the grocery store, cook only simple meals, and could only do light housework. |

How long these activity limits lasted also could be important to consider. Activity limits affected people on a daily basis throughout the **entire time they took the treatment** until they had a relapse and switched to other treatments.

**Chance of 10-day Hospitalization within 3 Months**

Treatments for multiple myeloma have varied in the number of patients who had **serious, but not permanent, complications related to brain functioning or their immune system in the first 3 months** of starting treatment.

**Brain-related complications** included:

- seeing things that are not real (hallucinations)
- seizures
- unable to move parts of the face or body
- temporary coma, during which people are unresponsive, cannot speak or follow basic commands

Signs of **immune system complications** included:

- fever, chills, difficulty breathing, irregular heartbeat, and severe pain.

These complications were serious enough for people to be **hospitalized for about 10 days**. During the hospital stay, they received multiple tests and supportive medical care, but these problems **went away within 2 months of being hospitalized**.

**Chance of complications that led to death within 3 months**

Some multiple myeloma treatments caused complications that led to people **dying within the first 3 months of treatment.**

Complications that led to death included infection, blood clots, and organ failure. In many cases, these conditions were successfully managed with intense medical treatment.

However, when thinking about potential treatment options for multiple myeloma in this survey, you will want to think about the chance of your friend having a complication that **leads to death even with medical treatment**.

**2. Example Choice Question**


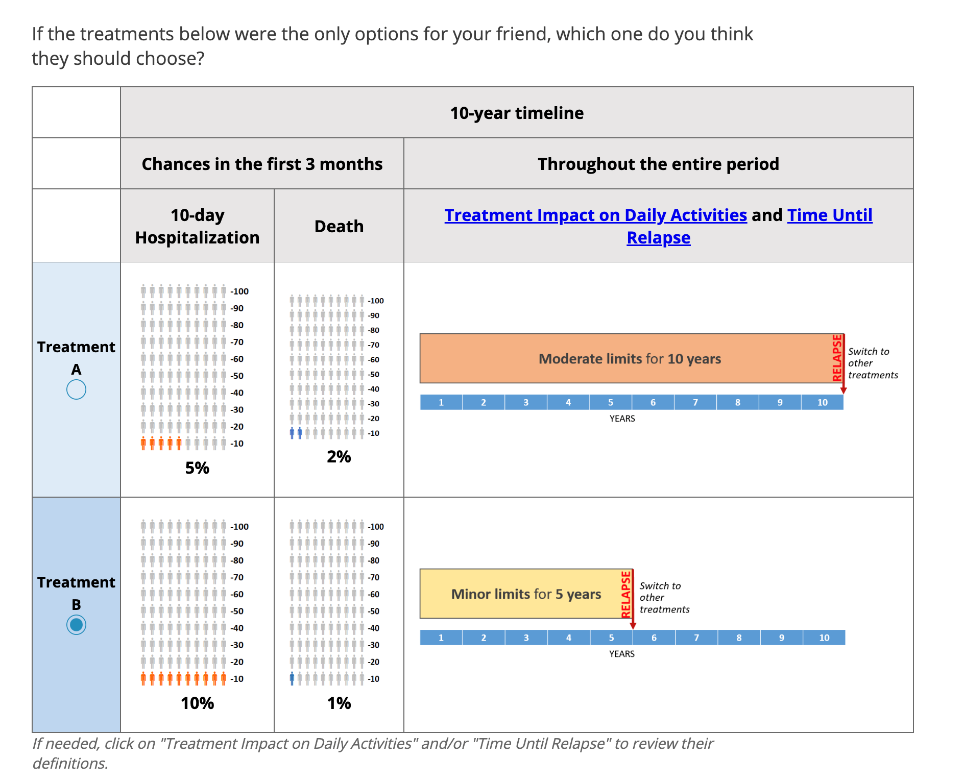
**Figure S1. Example DCE Question**

DCE: discrete-choice experiment

**3. Debriefing Questions**

After the DCE questions, respondents were asked to provide their feedback on the DCE in a series of five debriefing questions. The majority of respondents agreed or strongly agreed with each of the statements.

**DCE Debriefing Questions (N=176)**

|  | N (%) |
| --- | --- |
| Doctors and health care regulators should consider my responses in making their decisions. |  |
| Strongly disagree | 2 (1.1%) |
| Disagree | 2 (1.1%) |
| Neither agree or disagree | 28 (15.9%) |
| Agree | 67 (38.1%) |
| Strongly agree | 77 (43.8%) |
| I would make the same choices if I took the survey again. |  |
| Strongly disagree | 2 (1.1%) |
| Disagree | 3 (1.7%) |
| Neither agree or disagree | 20 (11.4%) |
| Agree | 83 (47.2%) |
| Strongly agree | 67 (38.1%) |
| Missing | 1 (0.6%) |
| I understood the treatment characteristics in the choice questions. |  |
| Strongly disagree | 1 (0.6%) |
| Disagree | 4 (2.3%) |
| Neither agree or disagree | 8 (4.6%) |
| Agree | 82 (46.6%) |
| Strongly agree | 81 (46%) |
| I had enough information to answer the choice questions. |  |
| Strongly disagree | 1 (0.6%) |
| Disagree | 8 (4.6%) |
| Neither agree or disagree | 12 (6.8%) |
| Agree | 81 (46%) |
| Strongly agree | 73 (41.5%) |
| Missing | 1 (0.6%) |
| I would recommend this survey to family and friends who qualify for this study. |  |
| Strongly disagree | 0 (0%) |
| Disagree | 2 (1.1%) |
| Neither agree or disagree | 14 (8%) |
| Agree | 86 (48.9%) |
| Strongly agree | 73 (41.5%) |
| Missing | 1 (0.6%) |

**4. Exploratory analysis of choice data**

Exploratory analysis of the choice data began with a conditional logit model with effect-coded variables for all levels in each attribute. This model specification imposed the fewest assumptions regarding the functional forms across preference weights within the study attribute levels but imposed simplifying assumptions on error terms. Conditional-logit models were run with continuous, dummy, and effects coding and were used to investigate potential interaction effects. Interactions between the attribute representing time to relapse and the attribute representing activity limitations were significant and included in the final model specification. There was not a statistically significant interaction between treatment-related adverse-event risks requiring hospitalization and mortality. The functional form for mortality risk levels was nearly linear while the functional form for hospitalization-risk levels was not. Based on the exploratory analysis, the model specification chosen was an effect-coded model with interaction terms for time to relapse and limitations on daily activities.

The choice data were analysed using a random-parameters logit (RPL) model which provides mean, sample-level relative preference weights for each attribute level. RPL models allow for estimating a distribution of tastes for each attribute level described by a preference-weight mean and standard deviation.

*Random-parameters Logit Results*

The relative mean preference weights from the RPL model (**Figure S1 and Table S1**), demonstrated that patients’ preferences were logically ordered for all attributes; that is, they preferred lower levels of risk to higher levels, more time until relapse to less time, and no or minor limits on daily activities over moderate limits. Holding treatment impact on daily activities constant, longer time until relapse was preferred to shorter time to relapse (all p-values <0.0001). At 10 years until relapse, preference weights for no limits, minor limits and moderate limits were statistically different from each other (no limits to minor limits: p=0.017; no limits to moderate limits: p<0.001; minor limits to moderate limits: p=0.002). At 5 and 3 years until relapse, preference weights for no limits and minor limits were not statistically different (p=0.701, p=0.143). However, at both time points, both no limits and minor limits were preferred to moderate limits (5 years no limits to moderate limits: p<0.001; 5 years minor limits to moderate limits: p<0.001; 3 years no limits to moderate limits: p=0.001; 3 years minor limits to moderate limits: p=0.032). Preference weights for the risk of hospitalization due to an adverse event for 10 days were insignificant until the risk level reached 20%. While 5% and 10% risk were not statistically different from each other (p=0.64), all lower risk levels (i.e. 0%, 5% and 10%) were statistically significantly different from 20% (0% to 20%: p=0.007; 5% to 20%: p=0.006; 10% to 20%: p=0.012). As expected, patients’ choices indicated a preference for lower risks of death in the next 3 months compared to higher risks. The p-value for the difference between 0% and 1% risk was p=0.050.

**Table S1. Preference Weights from the RPL Model (N=176)**

| Attribute | Level | | Coefficient | SE | P-value |
| --- | --- | --- | --- | --- | --- |
| **Parameter estimates** |  |  |  |  |  |
| Treatment Impact on Daily Activities and Time until Relapse | None | 10 years | 2.58 | 0.45 | <0.001 |
|  |  | 5 years | 0.67 | 0.22 | 0.002 |
|  |  | 3 years | -0.94 | 0.27 | 0.001 |
|  | Minor | 10 years | 1.69 | 0.33 | <0.001 |
|  |  | 5 years | 0.55 | 0.21 | 0.008 |
|  |  | 3 years | -1.43 | 0.29 | <0.001 |
|  | Moderate | 10 years | 0.30 | 0.28 | 0.291 |
|  |  | 5 years | -1.23 | 0.25 | <0.001 |
|  |  | 3 years | -2.18 | 0.38 | <0.001 |
| Chance in the first 3 months:  10-day Hospitalization | 0% | | 0.21 | 0.13 | 0.124 |
|  | 5% | | 0.17 | 0.12 | 0.184 |
|  | 10% | | 0.07 | 0.12 | 0.541 |
|  | 20% | | -0.44 | 0.14 | 0.002 |
| Chance in the first 3 months: Death | 0% | | 1.46 | 0.25 | <0.001 |
|  | 1% | | 1.03 | 0.20 | <0.001 |
|  | 5% | | -0.24 | 0.11 | 0.037 |
|  | 10% | | -2.25 | 0.37 | <0.001 |
| **Standard Deviations** |  | |  |  |  |
| Treatment Impact on Daily Activities and Time until Relapse | None | 10 years | 0.67 | 0.54 | 0.215 |
|  |  | 5 years | 0.35 | 0.58 | 0.545 |
|  |  | 3 years | 1.45 | 0.38 | <0.001 |
|  | Minor | 10 years | 0.99 | 0.50 | 0.048 |
|  |  | 5 years | -0.29 | 0.70 | 0.678 |
|  |  | 3 years | -0.97 | 0.41 | 0.017 |
|  | Moderate | 10 years | -2.72 | 1.72 | 0.114 |
|  |  | 5 years | -0.04 | 0.40 | 0.915 |
|  |  | 3 years | 0.56 | 0.63 | 0.373 |
| Chance in the first 3 months:  10-day Hospitalization | 0% | | 0.80 | 0.20 | <0.001 |
|  | 5% | | 0.36 | 0.31 | 0.244 |
|  | 10% | | 0.07 | 0.31 | 0.814 |
|  | 20% | | -1.23 | 0.52 | 0.018 |
| Chance in the first 3 months: Death | 0% | | 1.03 | 0.23 | <0.001 |
|  | 1% | | 0.97 | 0.21 | <0.001 |
|  | 5% | | 0.11 | 0.26 | 0.660 |
|  | 10% | | -2.12 | 0.48 | <0.001 |

Note: Attribute levels in this model were effect-coded with interaction terms for time to relapse and limitations on daily activities. With effect coding, the p-values are in reference to the mean preference weight for that attribute, and the preference weights for the attribute will sum to zero. The resulting coefficients are the sample-level relative mean preference weights for each attribute level included in the study, centered around zero. Within each attribute, a higher weight indicates a more preferred level. Moderate impacts with 10 years until relapse, 20% risk of hospitalization, and 10% risk of death are the omitted levels. The preference weights for the omitted levels were calculated as the negative sum of the coefficients for the other levels for that attribute. P-values for omitted levels are calculated using the delta method. SE= standard error

**Figure S1. Relative Preference Weights, RPL model (N=176)**


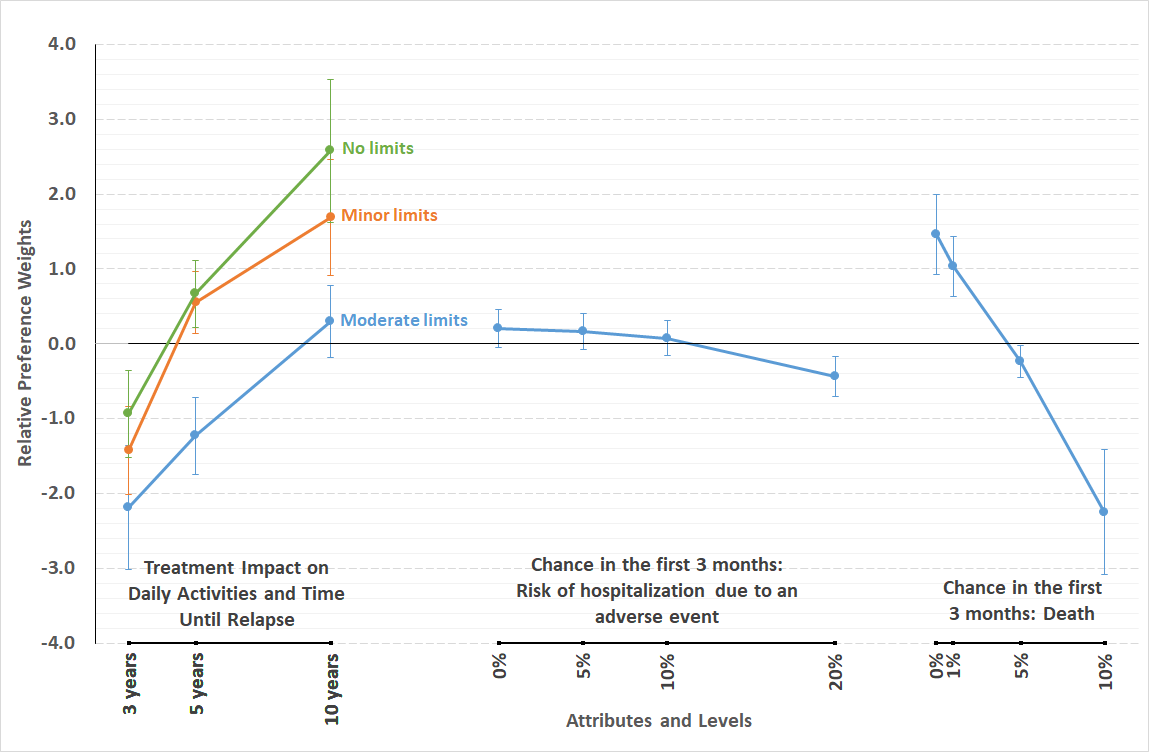


Error bars are the 95% confidence intervals around the mean. A higher relative preference weight indicates a more preferred outcome.
